# Trogocytosis in Unicellular Eukaryotes

**DOI:** 10.3390/cells10112975

**Published:** 2021-11-01

**Authors:** Kumiko Nakada-Tsukui, Tomoyoshi Nozaki

**Affiliations:** 1 Department of Parasitology, National Institute of Infectious Diseases, Tokyo 162-8640, Japan; 2 Department of Biomedical Chemistry, Graduate School of Medicine, The University of Tokyo, Tokyo 113-8654, Japan

**Keywords:** trogocytosis, phagocytosis, unicellular eukaryotes, *Entamoeba histolytica*, parasites, cross-dressing, intercellular communication

## Abstract

Trogocytosis is a mode of internalization of a part of a live cell by nibbling and is mechanistically distinct from phagocytosis, which implies internalization of a whole cell or a particle. Trogocytosis has been demonstrated in a broad range of cell types in multicellular organisms and is also known to be involved in a plethora of functions. In immune cells, trogocytosis is involved in the “cross-dressing” between antigen presenting cells and T cells, and is thus considered to mediate intercellular communication. On the other hand, trogocytosis has also been reported in a variety of unicellular organisms including the protistan (protozoan) parasite *Entamoeba histolytica*. *E. histolytica* ingests human T cell line by trogocytosis and acquires complement resistance and cross-dresses major histocompatibility complex (MHC) class I on the cell surface. Furthermore, trogocytosis and trogocytosis-like phenomena (nibbling of a live cell, not previously described as trogocytosis) have also been reported in other parasitic protists such as *Trichomonas*, *Plasmodium*, *Toxoplasma*, and free-living amoebae. Thus, trogocytosis is conserved in diverse eukaryotic supergroups as a means of intercellular communication. It is depicting the universality of trogocytosis among eukaryotes. In this review, we summarize our current understanding of trogocytosis in unicellular organisms, including the history of its discovery, taxonomical distribution, roles, and molecular mechanisms.

## 1. Introduction

Phagocytosis is a fundamental cellular process in eukaryotes. It is generally accepted that the last eukaryotic common ancestor emerged by internalization of an α-proteobacterium by a phagocytic archaeon, which leads to the mitochondrion, the multifunctional powerhouse of eukaryotes. Furthermore, phagocytosis is believed to be operative in early unicellular eukaryotes as a mechanism for feeding and for defending against predators [1]. Thus, phagocytosis is believed to have played significant roles during the evolution of eukaryotes [2]. In this context, all eukaryotes are, or were, at least some time in evolution, capable of bona fide phagocytosis. However, a majority of cells other than professional phagocytes in multicellular organisms are apparently and presumed to be non-phagocytic. A series of recent studies have revealed that phagocytosis is also present in non-professional phagocytes, and is presumed to play a broader range of roles in multicellular organisms.

While canonical phagocytosis is being demonstrated in a broad range of cell types in multicellular organisms, a new mode of phagocytosis, called trogocytosis, has gained attention. The word “trogo” in Greek means “to nibble” and implies ingestion by piecemeal, which is distinct from phagocytosis, in which a cell internalizes a prey not as pieces, but as a whole without disintegration. Besides morphological differences, the biological role of trogocytosis also seems to be distinct from that of canonical phagocytosis. For instance, trogocytosis has been demonstrated to be involved in a plethora of biological functions in multicellular eukaryotes, such as immune modulation, anti-cancer and anti-pathogen activities, neural homeostasis, embryogenesis, and transmission and propagation of infective agents. In immune cells, trogocytosis is involved in the “cross-dressing” between antigen presenting cells and T cells, and is thus considered to serve for intercellular communication. On the other hand, trogocytosis has been also reported in a variety of unicellular organisms. Trogocytosis has been demonstrated in *Entamoeba histolytica*, the protistan (protozoan) parasite, which ingests human T cell line by trogocytosis and acquires complement resistance and cross-dresses major histocompatibility complex (MHC) class I on the cell surface. Trogocytosis and trogocytosis-like phenomena (nibbling of a live cell, not previously described as trogocytosis) have been also reported in other parasitic protists such as *Trichomonas*, *Plasmodium*, *Toxoplasma*, and free-living amoebae. Thus, trogocytosis is conserved in diverse eukaryotic supergroups as the means of intercellular communication, thus, depicting the ubiquity among eukaryotes. In this review, we aim to show the ubiquity of trogocytosis in eukaryotes. We summarize our current understanding of trogocytosis in unicellular organisms, including history of discovery, taxonomical distribution, biological roles, and molecular mechanisms of trogocytosis.

## 2. Initial Discovery of Cell Nibbling in Unicellular Eukaryotes

Probably, the first report that described trogocytosis by a unicellular organism was made on a free-living amoeba, *Amoeba proteus* [3] (Table 1). In this report, *A. proteus* demonstrated an unusual ingestion behavior: it attached a paramecium, but ingested only half of the pray, leaving the other half uninternalized. Subsequently, it was also reported that *A. proteus* partly ingested the ciliate *Frontonia* [4] (note that all evidence was provided by illustrations, not photos). Later, fine microscopic images of cell nibbling of a live cell by *A. proteus* and another free-living amoeba, *Chaos carolinensis*, were documented [5]. Trogocytosis was also demonstrated in the slime mould, *Dictyostelium caveatum*, which internalized a sibling species *Dictyostelium discoideum* [6,7]. Since *Dictyostelium* can also ingest bacteria as prey, internalization of *D. discoideum* by *D. caveatum* was presumed to play a role other than nutrient acquisition. In parallel, Culbertson and colleagues conducted a series of studies on the nibbling behavior of *Naegleria fowleri* (called *N. aerobia* then). In their studies, trogocytosis-like events by two species (considered then as two morphologically distinguishable strains of a species) of soil amoebas, *N. fowleri* HB-1 and *Hartmannella-Acanthamoeba* A-1 [8,9], were described. When those amoebae were inoculated into guinea pigs, it was demonstrated by pathological examination that the amoebae in the thrombi internalized erythrocytes only halfway (i.e., trogocytosis) [9]. Later, trogocytosis of a mouse embryonic cell by *N. fowleri* was also demonstrated by immunofluorescence assay and electron microscopy [10]. In this report, the word “trogocytosis” was used for the first time. Trogocytosis was also described in the enteric parasitic protist, *E. histolytica*, although the phenomenon regained attention due to presumed contribution to pathogenesis, but had not been referred as trogocytosis [11,12,13,14,15] until recently [16].

## 3. General Role of Trogocytosis in Eukaryotes

Several biological roles of trogocytosis have been experimentally demonstrated in unicellular eukaryotes, while other roles were only suggested: feeding/nutrient acquisition, defense/self-protection, pathogenicity, self-nonself discrimination, and intercellular communication. Among these roles, trogocytosis (or trogocytosis-like process) is evidently involved in the three former functions in many protozoa. Also, intercellular communication was demonstrated between *E. histolytica* and human cells. Self-nonself discrimination was experimentally shown in a *Dictyostelium* species, however, the molecular mechanism is not fully elucidated.

### 3.1. Feeding/Nutrient Acquisition

The internalized preys can be used as nutrient source in both free-living and parasitic amoebas. Several studies demonstrated degradation of ingested materials [3,4,25,26,27,28,29]. However, a conundrum remains unsolved: what is the advantage of trogocytosis, i.e., partial ingestion of the prey? Possible reasons include thrift: a less amount of membrane (and thus lipids) and energy are used for trogocytosis than phagocytosis. This is quite against intuition because trogocytosis of target cells such as *Paramecium*, which has firm surface cytoskeleton composed of epiplasmin and actin, and surface cilia connected to the cytoskeleton [30,31,32], requires high energy and is not energetically economical. It is also of note that apoptotic cells are less elastic than live cells [33]. Another possible advantage of trogocytosis over phagocytosis is that trogocytosis allows selective acquisition of components such as membranes and exclusion of toxic substances such as oxygen and nitrogen radicals in phagosomes from professional phagocytes.

### 3.2. Pathogenicity

Trogocytosis of unicellular organisms has been often studied in the context of pathogenicity. Trogocytosis is involved in pathogenicity and virulence in at least three ways: 1. direct killing of target cells for cell and tissue damage; 2. elimination of immune cells; 3. generation of niche for symbiosis and parasitism; and 4. acquisition of host molecules for mimicry (see Section 3.4). It has been described in many free-living and parasitic protists such as *Naegleria*, *Hartmannella-Acanthamoeba*, and *Entamoeba* that trogocytosis was clearly implicated in virulence mechanisms [8,9,13,16]. The involvement of proteases in the intersection of trogocytosis and pathogenicity was shown in *E. histolytica*. Trogocytosis of live Jurkat cells by *E. histolytica*, but not phagocytosis of dead Jurkat cells, was inhibited by a cysteine protease inhibitor E-64 [34]. Trogocytosis of *Trichomonas vaginalis* trophozoite by a neutrophil was also inhibited by an elastase inhibitor, AEBSF, while the inhibitor did not affect phagocytosis of the dead parasite [35]. In both organisms, proteases are involved in host cell degradation and tissue destruction [36,37,38,39,40,41]. Furthermore, contribution of proteases to pathogenicity was also demonstrated in *Naegleria* and *Acanthamoeba* [42,43,44,45]. It needs to be elucidated in those organisms whether protease(s) are also involved in trogocytosis per se. Furthermore, it is necessary to elucidate how proteases are involved in trogocytosis: surface receptor processing, inflammasome formation, and downstream signaling.

### 3.3. Self-Nonself Discrimination

Self-nonself discrimination is often used in the context of immune recognition and immunological tolerance. However, there is also a significant numbers of cases in which the term was used in a broader sense with the representative case being cannibalism between close species of the free-living eukaryotes [46,47,48,49]. Trogocytosis has been suggested to play a role in self-nonself discrimination in the social amoeba *Dictyostelid* species. They are known to generate multicellular fruiting body under starvation conditions. When two different species are mixed, each species is segregated from each other and forms an independent fruiting body. However, in case where *D. caveatum* was mixed with *D. discoideum*, only the fruiting body of *D. caveatum* was formed [6] as *D. caveatum* ingested and killed *D. discoideum* by trogo- (and phago-) cytosis [6,7]. *D. caveatum* also trogocytosed themselves, suggesting that self-nonself discrimination mechanisms were selectively lost in this species [50]. The gene involved in self-recognition was identified in *Dictyostelid* [51,52]. *Dictyostelium*-specific polymorphic (among *D. discoideum* strains) transmembrane proteins, TgrB1/TgrC1 (Tgr = Tiger, Transmembrane, IPT, IG, E-set, Repeat protein), serve as a ligand-receptor set unique to each strain [52,53]. Importantly, the TgrB1/TgrC1 system is conserved in a limited group of *Dictyostelium* species, and neither of the proteins is conserved in *D. caveatum* [54]. Although further study is necessary to better understand the role of trogocytosis per se in the potential self-nonself discrimination mechanisms, *D. caveatum* is a unique example to understand the role of trogocytosis in kin discrimination. Kin discrimination of *Entamoeba* was also reported, however, neither responsible genes for self-recognition nor its relationship with trogocytosis has been identified [55].

### 3.4. Intercellular Communication

It has been demonstrated in immune cells that phagocytes receive surface proteins from target cells by trogocytosis, which are subsequently displayed on the surface of the phagocytes. This phenomenon is called cross-dressing and is known in a broad range of cell type pairs including natural killer (NK) cells, basophils, and T cells expressing an artificial chimeric antigen receptor (CAR) (CAR T cells) [17,56,57,58]. NK cells are known to dress MHC II, which is derived from dendritic cells (DC), and natural killer group 2 membrane D ligand (NKG2DL) from T cell lymphoma cells. As NK cells do not express co-stimulatory molecules necessary for MHC II antigen presentation, the removal of MHC II from DC and cross-dressing on NK cells causes reduction of the MHC II on DC and suppresses antigen presentation by DC [17]. NKG2DL on T cell lymphoma cell stimulates NKG2D on the NK cell to activate effector activity, leading to killing of T cell lymphoma cells by activated NK cells. NK cells cross-dressing NKG2DL derived from T cell lymphoma cells are killed by other NK cells [56]. This may be considered as one of the escape mechanisms of T cell lymphoma. Basophils were also demonstrated to acquire MHC II from DC by trogocytosis. Basophils then conducts antigen presentation to naïve CD4^+^ T cells and also provides IL-4 to promote differentiation into Th2 cells [57]. This Th2 reaction is considered to be involved in basophil-mediated allergy. Finally, CAR T cells receive CD19, a B cell antigen, from B cell lymphoma cells by trogocytosis and cross-dress CD19 on CAR T cells [58]. This cross-dressing of CD19 on CAR T cells causes fratricide of CAR T cells and reduction of CD19 on the B lymphoma cells, which eventually gives rise to tumor relapse. It was demonstrated that *E. histolytica* acquired complement resistance via trogocytosis of Jurkat T cells. Importantly, complement resistance was not gained by phagocytosis of dead cells [59]. It has been demonstrated in *Plasmodium falciparum*, that complement resistance is achieved by acquisition of complement inhibitory molecules CD55 and CD59 on the parasite or infected erythrocytes [60]. This suggests that *E. histolytica* also acquire CD55 and/or CD59. These molecules are GPI-anchored proteins. It was shown that transfer of GPI-anchored protein appears to occur directly between live cells and via exosomes [61,62,63]. Contrary, in *E. histolytica*, it was reported that the transfer of complement resistance depends on actin and direct contact between cells. It was also shown that *E. histolytica* cross-dresses transmembrane domain-containing MHC I, derived from Jurkat cells [59]. These observations may suggest a new paradigm wherein trogocytosis is involved in immune evasion of eukaryotic pathogens [64].

## 4. Trogocytosis in Parasitic Protists

Among unicellular organisms, trogocytosis and trogocytosis-like processes have been recently reported in a wide range of parasitic protists, including *E. histolytica*, Excavata including *Giardia intestinalis*, and *T. vaginalis* and Apicomplexan (*Plasmodium falciparum* and *Toxoplasma gondii*) (Table 1). If trogocytosis is a shared common mechanism among eukaryotes, we might find similar cell nibbling behavior in other protists than those so far reported. Among them, trogocytosis in *E. histolytica* is the best studied, and will be described in a later Section 5.

### 4.1. Trichomonas Vaginalis

*T. vaginalis* mainly colonizes the vagina and causes one of the most common sexually transmitted diseases worldwide, trichomoniasis [65]. *T. vaginalis* causes symptoms in humans by its cytopathic effects on host cells, including phago- and trogocytosis, induction of inflammation, and affecting microbiota [18,66]. *T. vaginalis* displays multiple morphologically discernible developmental stages, among which the amoeboid form is capable of phago- and trogocytosis [19,66]. It is of note that escape from complement attack is one of the essential immune evasion mechanisms of *T. vaginalis* [67,68]. *T. vaginalis* acquires CD59 from mouse erythrocytes by cross-dressing [69]. CD59 is a GPI-anchored membrane protein and protects the cell from complement attack by disturbing the formation of membrane attack complex. Although trogocytosis was not described per se, nibbling of live genitourinary epithelial cells by *T. vaginalis* was demonstrated [70]. It is conceivable that *T. vaginalis* utilizes trogocytosis for immune evasion by cross-dressing CD59 from mouse erythrocytes.

### 4.2. Giardia Intestinalis

Another anaerobic parasitic protist is *G. intestinalis*, which resides in human and animal small intestines. *G. intestinalis* was previously believed to internalize extracellular nutrients and essential factors from the extracellular milieu exclusively via fluid-phase and receptor-mediated endocytosis. Thus, it was considered that *G. intestinalis* lacks the capacity to carry out phagocytosis, until it has been recently demonstrated [71]. It was shown that *G. intestinalis* trophozoite generates pseudopods and forms phagocytic cup and phagosome to internalize bacteria, yeasts, and polystyrene or carboxylated microspheres [71]. It was demonstrated that internalization occurred through the entire cell surface, but, more frequently through the ventral flagella’s exit, where clathrin is abundant and receptor mediated endocytosis is operated [72]. Although neither nibbling nor trogocytosis per se was reported, it is conceivable that *G. intestinalis* is also capable of trogocytosis or trogocytosis-like internalization. Similar to *E. histolytica* and *T. vaginalis*, *G. intestinalis* is also attacked by complement. However, the mechanisms of complement resistance in *G. intestinalis* remains elusive [73,74].

### 4.3. Trypanosoma and Leishmania

*Trypanosoma* and *Leishmania* belong to the Kinetoplastida in the super group of Excavata, which *Trichomonas* and *Giardia* also belong to, and include a number of species that can cause diseases in humans and animals. The *Trypanosoma brucei* group causes African sleeping sickness in humans and nagana in cattle, while *Trypanosoma cruzi* causes American trypanosomiasis, Chagas’ disease, in humans, and is endemic in Central and South America [75,76]. A complex of *Leishmania* species is responsible for visceral, cutaneous, and mucocutaneous leishmaniasis in human worldwide [77,78,79]. It was demonstrated that *T. cruzi* epimastigotes (the insect stage, living in the insect mid- and hindgut) and amastigotes (the intracellular mammalian stage, living in the host cell cytoplasm) [80,81,82] display the cytostome-cytopharynx complex, which resembles elongated trogo- and phagosomes. The cytostome-cytopharynx complex is a structure that extends from a position adjacent to the flagellar pocket located at the root of the flagellum, to the distal end in the posterior near the nuclear periphery in the cell. The cytostome-cytopharynx seems to be unique to *T. cruzi* and has not been demonstrated in either *T. brucei* or *Leishmania* [20,21,83,84,85]. In *T. brucei* and *Leishmania*, endocytosis exclusively occurs at the flagellar pocket [21,22], whereas in *T. cruzi* endocytosis occurs at both the flagellar pocket and the cytostome. Since *T. cruzi* amastigotes reside in the host cell cytoplasm, amastigotes can directly internalize the cytoplasmic components at the flagellar pocket. It was demonstrated that endocytosis at the flagellar pocket is clathrin-dependent, whereas endocytosis at the cytostome is clathrin-independent. However, it remains elusive if *T. cruzi* amastigotes can ingest the host cell membranes and the membrane-bound organelles (e.g., ER, Golgi, endosomes, and mitochondria) via the cytostome.

### 4.4. Plasmodium

Five *Plasmodium* species, which belong to the Apicomplexa, cause malaria in humans and are responsible for over 0.45 million mortality annually among mostly children of <5 years old in sub-Saharan Africa [86]. Intracellular malaria parasites are segregated from the host cells by two layers of membranes [the parasitophorous vacuole (PV) membrane derived from the host cell and the parasite’s plasma membrane] in the host nucleated and anucleated cells. Therefore, malaria parasites are not expected to ingest host components. However, there is a line of evidence suggesting the presence of phago- or trogocytosis-like phenomena. The structure called the cytostome, which was also described in *T. cruzi* as above, was well documented in the blood (erythrocytic)-stages of *P. falciparum*, which need to utilize hemoglobin present in the erythrocyte cytosol, for growth [23,87,88]. *P. falciparum* displays the cytostome during an actin-dependent invagination of the erythrocyte cytoplasm into the parasite. The cytostome morphologically resembles the structure that forms during trogocytosis in other organisms and may be considered to be the structure related to a trogocytosis-like process. However, a unique electron-dense ring structure localized at the neck of the cytostomal invagination appears to be unique to *Plasmodium*, while a narrow tube which connected the cytostome with the food vacuole, called cytopharynx, may be functionally homologous to the tubular bridge found between a mature trogosome and primary (newly formed) trogosome frequently found in *E. histolytica*. [84,89,90,91,92]. Note that the term “trogosome” is defined as the endomembrane endosome-like system formed by trogocytosis, equivalent to the phagosome formed by phagocytosis, but most likely compositionally different from phagosomes and generated in a distinct molecular mechanism from phagosomes. It was shown that the cytostome-cytopharynx is eventually pinched off from the PV membrane and the erythrocyte cytoplasmic content is internalized to be decomposed in the food vacuole [91]. Since partial ingestion of live prey is the definition of trogocytosis, the cytostome-cytopharynx related phenomenon resembles trogocytosis. For instance, a subsequent association of Rab5A and PtdIns3P binding proteins (a FYVE domain containing protein, FCP, and Atg18) with the food vacuole, in the course of cytostome and trogosome formation, and inhibition of cytostome and trogosome formation by a PI3K inhibitor [24,93,94,95] are shared mechanisms by both of the processes. It has not yet been demonstrated that *P. falciparum* is able to cross-dress a protein that originated from the PV membrane.

### 4.5. Toxoplasma Gondii

*Toxoplasma gondii*, which also belongs to the Apicomplexa, causes toxoplasmosis, the most widespread zoonotic parasitic disease in humans [96]. One-third of the world human population is reported to be infected with *T. gondii*. *T. gondii* infection is often presented without serious symptoms in healthy individuals; however in immunocompromised individuals, it potentially leads to lethal encephalitis [97]. Similar to *Plasmodium*, *T. gondii* lives in the PV of nucleated cells. The endocytosis-like phenomenon, and a unique cellular structure associated with the phenomenon, the micropore, which is a characteristic electron-dense ring structure at the neck of the invagination in tachyzoites and bradyzoites [98], have been well documented. Different from the cytostome of *P. falciparum*, *T. gondii* apparently does not internalize the PV membrane by the micropore [98]. This observation was contradicted by recent studies [99,100,101], in which it has been shown that the micropore can pinch off the PV membrane. Furthermore, it was proposed that *T. gondii* exploits a trogocytosis-like pathway to engulf host Rab small GTPases-marked Golgi or Golgi-associated vesicles, which *T. gondii* closely resides to, in order to gain sphingolipids [102,103]. It was further demonstrated that the Golgi-associated vesicles are first sequestered to the intervascular network (IVN), the membrane tubules invaginated from the PV membrane, and subsequently internalized by the parasite [104]. The phenomenon represents one good example of intercellular information exchange in intracellular protozoa. However, it needs to be further validated at the molecular level whether the phenomenon in *T. gondii* is functionally comparable to trogocytosis or a trogocytosis-like process because the event in *T. gondii* relies on microtubules but not actin, which is different from trogocytosis in other organisms.

## 5. Molecular Mechanisms of Trogocytosis in *E. histolytica*

**Figure 1:**
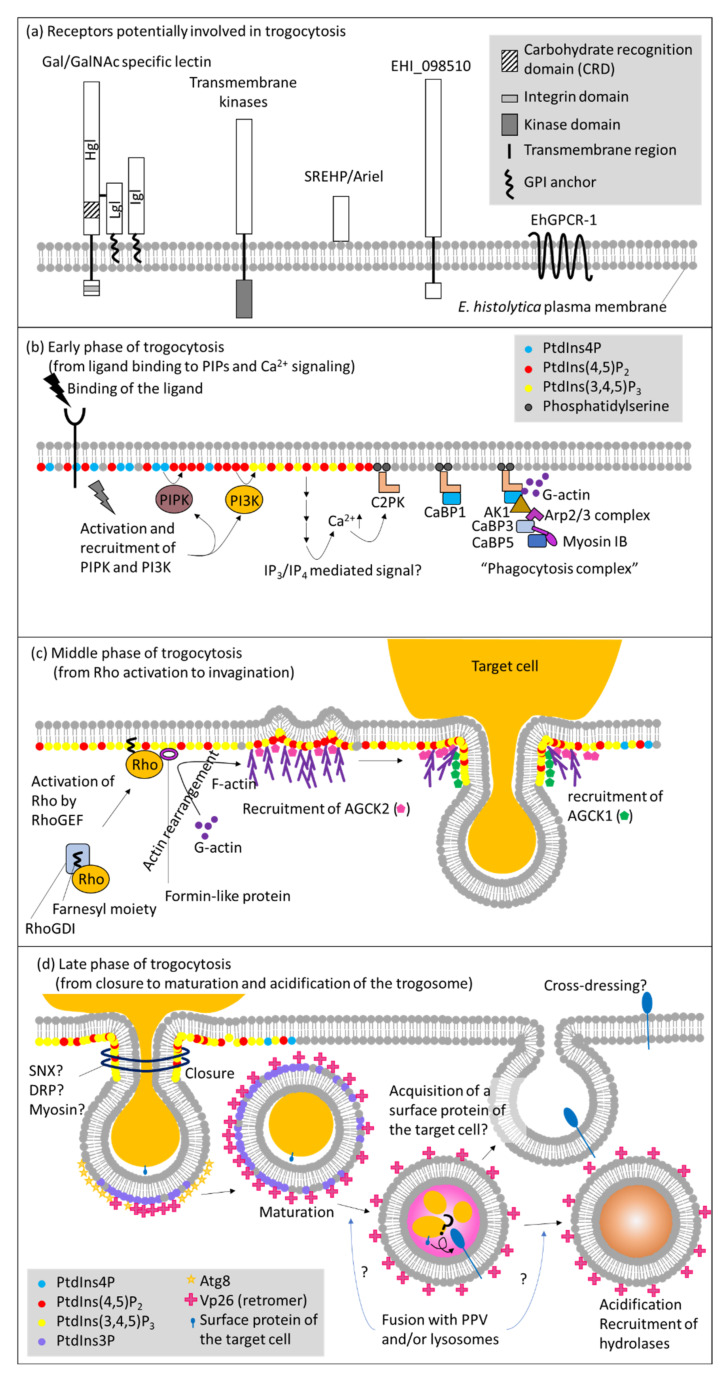
Molecular mechanisms of trogocytosis in *E. histolytica*. Schematic representation of molecules known or predicted to be involved in trogocytosis in *E. histolytica*. (**a**) Receptors that are presumed to be involved in the initiation of trogocytosis, by analogy to phagocytosis. A series of major events and molecules involved at each step during the (**b**) early(from ligand binding to PIPs and Ca^2+^ signaling), (**c**) middle (from Rho activation to invagination), and (**d**) late (from closure to maturation and acidification of the trogosome) phases of trogocytosis, respectively. Abbreviations: AK, atypical kinase; Arp2/3 complex, actin related protein 2/3 complex; C2PK, C2 domain-containing protein kinase; CaBP, calcium binding protein; DRP, dynamin-related protein; GPCR, G protein-coupled receptor; GPI, glycosylphosphatidylinositol; IP3, inositol trisphosphate; IP4, inositol tetrakisphosphate; PI3K, phosphatidylinositol 3-kinase; PIPK, phosphatidylinositol phosphate kinase; PPV, prephagosomal vacuole; PtdIns, phosphatidylinositol; RhoGDI, Rho guanine nucleotide dissociation inhibitor; RhoGEF, Rho guanine nucleotide exchange factor; SNX, sorting nexin; SREHP, serine rich *Entamoeba histolytica* protein.

Among unicellular eukaryotes, trogocytosis have been best studied in *E. histolytica* at the molecular level [16,29,34,105,106]. Proteins and lipids involved in trogocytosis are largely conserved between human and *E. histolytica*. So far, only one protein, AGC kinase 1 (AGCK1) from *E. histolytica*, clearly differentiates trogocytosis and phagocytosis [105,107]. However, a series of events that exclusively and selectively occur in trogocytosis, but not in phagocytosis, and the underlying mechanisms, need to be demonstrated to better understand the physiological significance of trogocytosis. In this part, we divided the sequential events that occur in trogocytosis into 6 major phases: (1) target recognition via a receptor on the plasma membrane, (2) triggering of phosphatidylinositol (PtdIns) and calcium signaling on the plasma membrane; (3) recruitment of effectors; (4) actin rearrangement leading to eventual internalization of the target; (5) closure of the trogosome; and (6) acidification and maturation of the trogosome. We summarized below the current knowledge on the key events that occur during trogocytosis in *E. histolytica* (Figure 1 and Table 2). Although we assume that a majority of the events are common for trogocytosis and phagocytosis, this assumption needs to be experimentally proven in future (Table 2).

### 5.1. Receptors and Downstream Signaling

Trogocytosis and phagocytosis are induced by ligand-receptor binding. In model organisms, a variety of ligand-receptor systems involved in direct recognition of pathogens are known, including pathogen-associated molecular patterns (PAMPs) and pattern-recognition receptors (PRRs). It was well established that PRRs such as Dectin-1, Mincle, MCL, and DC-SIGN trigger phagocytosis [108]. Alternatively, pathogens are first decorated with immunoglobulins or complement C3bi and subsequently recognized by Fc-γ and CR3 receptor, respectively [109]. Apoptotic cells are also directly or indirectly (via phosphatidylserine-binding proteins) recognized by receptors for externalized phosphatidylserine [110]. In *E. histolytica*, surface receptors had been previously identified before the concept of trogocytosis regained attention. Thus, in a strict sense, most of the receptors were demonstrated to be involved in phagocytosis, but not trogocytosis per se. Such receptors include transmembrane kinases (TMKs) [TMKB3-96 (PATMK), TMKC-39, and TMKB1-9], serine rich *Entamoeba histolytica* protein (SREHP)/Ariel, EHI_098501, and G protein-coupled receptor (EhGPCR1) [111,112] (Figure 1a).

It was demonstrated that Gal/GalNAc-specific lectin (Gal lectin), composed of the transmembrane domain-containing heavy subunit and the GPI-anchored light and intermediate subunits, is involved in trogocytosis using a specific antibody against the heavy subunit [16]. However, Gal lectin is not specifically involved in trogocytosis, but also phagocytosis. Gal lectin is involved in the adhesion to bacteria, erythrocytes, live CHO cells, live and dead Jurkat cells, and mucin [16,113,114,115,116,117,118,119]. However, downstream signaling directly elicited by the binding of a ligand to Gal/GalNAc specific lectin is not well understood. The integrin-like domain in the 41 a.a.-long cytoplasmic region of the heavy subunit is assumed to play a role in inside-out signaling and adherence to the target [120]. Besides, neither recognizable domains nor potential binding proteins have been identified for the lectin, leaving no clue for downstream signaling events [121,122,123]. It was shown that expression of GFP fused to the transmembrane domain and the C-terminal cytoplasmic region of the heavy subunit caused dominant negative effect on the adhesion to CHO cells, and liver abscess formation in the animal model. However, ingestion of erythrocytes, complement resistance, and cytolysis toward mammalian cells were unaffected [120], which were counter-intuitive, but maybe indicative of attachment and downstream events occur independently. Involvement of TMKs, SREHP/Ariel, EHI_098501, and EhGPCR1 in trogocytosis is not demonstrated, while they were shown to be engaged in phagocytosis of erythrocytes, mammalian cells, and bacteria [111,112].

We recently identified a potential trogocytosis-specific isotype of the heavy subunit of the lectin. This protein is apparently involved in the trogocytosis of fresh human erythrocytes, and, to a lesser extent, phagocytosis of aged or damaged erythrocytes (unpublished).

### 5.2. Phosphatidylinositol, Calcium Signaling, and Protein Kinases

The role of phospholipids and calcium signaling on the plasma membrane in trogocytosis and phagocytosis has been well documented [16,105,124,125,126]. Wortmannin, a phosphatidylinositol 3-kinase (PI3K) inhibitor was shown to inhibit trogocytosis and phagocytosis [16,124,127] (Figure 1b).

Neither the compartment of Ca^2+^ storage nor the mechanisms for Ca^2+^ release in *E. histolytica* has been clearly demonstrated. However, it was shown that inositol 1,4,5-trisphosphate (IP_3_) and inositol 1,3,4,5-tetrakisphosphate (IP_4_) likely regulate Ca^2+^ mobilization [128,129]. These second messengers are generally generated from phosphatidylinositol 4,5-bisphosphate [PtdIns(4,5)P_2_] by the action of phospholipase C (PLC), although homologues for PLC and IP_3_ receptor are lacking in *E. histolytica* [125,129]. It may be possible that an unknown phospholipase other than PLC cleaves PtdIns(4,5)P_2,_ or an alternative pathway exists to generate IP_3_ and IP_4_ like what was reported in *Dictyostelium* [130]. The absence of predictable orthologs for IP_3_ receptor is observed in several protozoa, and is currently attributed to the low levels of conservation at the primary sequences, as shown in *Trypanosoma cruzi* [131,132]. Later, it has been shown that *Dictyostelium* has an alternative pathway to generate IP_3_ from Ins(1,3,4,5,6)P_5_ [133]. C2 domain containing protein kinase a calcium-binding protein, C2PK, is translocated to the plasma membrane and binds to phosphatidylserine in a Ca^2+^ dependent manner [124]. C2PK subsequently recruits Ca^2+^ binding protein 1 (CaBP1) and other effectors including CaBP3, CaBP5, atypical kinase 1 (AK1), Arp2/3 complex, and myosin IB, which consist the “phagocytosis complex” [124,126,134]. Since both C2PK and CaBP1 are actin-binding proteins [124], calcium signaling plays a pivotal role in actin dynamics during trogocytosis and phagocytosis. The involvement of C2PK in trogocytosis has been shown: expression of kinase dead C2PK caused defect in trogocytosis and phagocytosis. It is conceivable to speculate a possibility that the components of the phagocytosis complex may vary between trogocytosis and phagocytosis.

Among a number of phosphatidylinositol 3,4,5-trisphosphate [PtdIns(3,4,5)P_3_] binding proteins, AGCK1 was shown to be exclusively involved in trogocytosis [105,135] (Figure 1c). AGCK1 and another isotype of AGCK, AGCK2, were identified by affinity pull down using PtdIns(3,4,5)P_3-_immobilized beads in our attempt to isolate PtdIns(3,4,5)P_3_ binding proteins [105]. It was clearly demonstrated that AGCK1 was exclusively involved in trogocytosis, but not phagocytosis, by reverse genetic studies in which expression of AGCK1 or AGCK2 was specifically repressed by small antisense RNA-medicated transcriptional gene silencing, or when kinase dead mutants were expressed. In contrast to the specific role of AGCK1 in trogocytosis, AGCK2 was shown to be involved in a broad range of endocytic processes including fluid-phase endocytosis, trogocytosis, and phagocytosis. AGCK1 and AGCK2 also showed distinct recruitment and localization profiles during trogocytosis. Prior to trogocytosis, AGCK2 was localized at the attachment site of the plasma membrane. Upon initiation of trogocytosis, AGCK2 was concentrated at the invagination site in the plasma membrane proximal region. At the same time, AGCK1 was localized at the tunnel structure that connected the partially ingested target cell and the newly formed (not yet enclosed) trogosome (called the trogocytic tunnel). Generation of the trogocytic tunnel structure appears to be the key event of trogocytosis. AGCKs are generally activated downstream of class I PI3K [136]. The *E. histolytica* genome encodes 24 AGCKs and 6 class I PI3Ks [125,137,138]. It is important in future studies to determine whether other AGCKs are also specifically or universally involved in trogocytosis and phagocytosis, and which class I PI3Ks activate such AGCKs. Furthermore, two Rho guanine nucleotide exchange factor (RhoGEFs) and one formin domain containing protein were identified as PtdIns(3,4,5)P_3_ binding proteins [135], which also reinforces the notion that PtdIns(3,4,5)P_3_ is involved in cytoskeleton reorganization (see below).

### 5.3. Cytoskeletal Reorganization via Rho Small GTPases

Rho small GTPases are known to play a pivotal role in actin cytoskeleton reorganization (Figure 1c) and may be key players to differentiate trogocytosis and phagocytosis. In model organisms, it is known that three representative Rhos – RhoA, Rac, Cdc42, and RhoGEF, which is activated via receptor signaling, are involved in the formation of stress fiber, lamellipodia, and filopodia [139,140]. Lamellipodia formation is associated with and is necessary for trogocytosis and phagocytosis. In human macrophages, it was demonstrated that activation of RhoA, instead of Rac, enhanced nibbling (trogocytosis) and reduced removal of necroptotic cells [141]. *E. histolytica* possesses 19 Rho small GTPases. Although there are only limited studies on each Rho available, specific roles of some isotypes were demonstrated. For example, EhRacA is involved in phagocytosis of erythrocytes, but not in fluid phase endocytosis [127], whereas EhRacG is involved in cytokinesis and uroid formation [142]. EhRho1 contributes to phagocytosis and formation of membrane blebs [143,144]. Thus, it is conceivable that specific Rhos are differentially involved in trogocytosis and phagocytosis in *E. histolytica*.

### 5.4. Closure of Trogosomes

To complete internalization of the prey, sealing of the trogocytic cup and pinching-off of the newly formed trogosome is needed (Figure 1d). Generally, dynamin or BAR domain-containing sorting nexin (SNX) is responsible in pinching-off of the plasma membrane invagination. Dynamin-1 and LST4 (SNX9) were demonstrated during trogocytosis in *C. elegans* [145]. *E. histolytica* possesses 4 dynamin-related proteins (Drps), two of which are in the cytosol while the other two are in the nucleus [146]. While these two cytosolic Drps are involved in mitosome fission, they may also be involved in trogosome pinch-off. *E. histolytica* also possesses 30 BAR domain proteins. However, no SNX protein with dual BAR and PX domains is present [29], leaving a potential involvement of BAR domain proteins in trogosome formation an open question. It was previously shown that myosins are involved in the closure of the phagosome in macrophages [147]. *E. histolytica* has a single myosin, myosin ⅠB, and its role in cooperation with CaBP3 in phagosome closure was demonstrated [148]. In phagocytosis, CaBP1 recruits AK1, which further recruits Arp2/3 complex. The Arp2/3 complex binds to CaBP3, which is a myosin ⅠB binding protein, in a Ca^2+^ dependent manner. This series of events localizes myosin ⅠB and CaBP3 at the site of phagosome closure [148]. Thus, it is plausible that CaBP3 and myosin ⅠB also play a similar role in trogocytosis [148].

### 5.5. Maturation of Trogosomes vs. Phagosomes

It is not well understood if molecules involved in the maturation of the trogosome differ from those in phagosome maturation. Several key molecules are apparently shared in both trogocytosis and phagocytosis, such as Vps26 (retromer), Atg8, and PtdIns3P [28,29,149,150] (Figure 1d). It is worth noting that the prephagosomal vacuole (PPV) may be more closely associated with phagocytosis than trogocytosis. PPV was first identified as a large vacuolar compartment that emerged upon ingestion of human erythrocytes [26]. PPV contains various hydrolases and fuses with the primary phagosome to deliver digestive proteins [26]. PPVs were more frequently observed when *E. histolytica* trophozoites were incubated with dead mammalian cells to allow phagocytosis than when incubated with live cells (Nakada-Tsukui, unpublished observation). However, the precise role of PPV in phagocytosis remains undetermined. Cross-dressing between immune cells, described above, is observed only when a live target cell is ingested by the recipient cell via trogocytosis [17,56,57,58]. During trogocytosis and cross presentation, membrane proteins that have been derived from the host cell plasma membrane via trogocytosis, need to retain integrity and topology to be properly cross presented on the recipient’s plasma membrane. In contrast, the content of phagosomes cannot be selectively degraded. Thus, it is conceivable that the maturation process differ between trogosomes and phagosomes.

It has been well established that a panel of hydrolases including proteases, peptidases, and glycosidases are recruited to phagosomes [151,152,153,154]. However, it remains to be determined if these hydrolases are specifically recruited to trogosomes vs. phagosomes. In accordance with the premise mentioned above, it is probably so. It has been recently shown that trogocytosis, but not phagocytosis, was inhibited by a cysteine protease inhibitor [34]. A similar observation was demonstrated in neutrophils, in which an elastase inhibitor selectively inhibits trogocytosis [35]. It is not well understood how proteases are involved in trogocytosis, other than degradation of ingested substances, and why inhibition of protease activity selectively represses trogocytosis. Proteases may be involved in the deformation of the prey, which is needed for trogocytosis.

### 5.6. Physiological Role of Trogocytosis in E. histolytica

*E. histolytica* exploits trogocytosis for multiple purposes. First, *E. histolytica* uses trogocytosis for nutrients, presumably membrane lipids, from the host cells and other eukaryotic prey. The advantages of trogocytosis for nutrient acquisition are described above (see Section 3.1). Second, *E. histolytica* acquires complement resistance via trogocytosis, which was demonstrated in an experiment using Jurkat T cells. Complement resistance was not gained by phagocytosis of dead cells [59]. Although the molecule responsible for the transfer of complement resistance remains unknown, dressing of MHC I, CD59 or another complement inhibitory molecule is likely responsible for this phenomenon. In this context, trogocytosis is integrated in parasitism, immune evasion, and pathogenesis. Finally, although it remains unclear if trogocytosis is a measure of self-nonself discrimination, the topic seems worth pursuing.

## 6. Conclusions and Future Perspective

As summarized in this review, trogocytosis is a widely conserved mechanism in eukaryotes including free-living and parasitic protists. It is likely that trogocytosis will be demonstrated in a broader range of eukaryotes and the functional diversity of trogocytosis other than nutrient acquisition, pathogenicity, immunity, self-nonself discrimination, and immune evasion, shall be demonstrated in life. The link between trogocytosis and self-nonself discrimination discovered in free-living and parasitic eukaryotes has led us to a key question: “what is the self?”. If trogocytosis and cross-dressing modifies the self without a trace of change in the genome, the diversity of life on Earth, in the context we currently understand through genetic information, is largely underestimated. Furthermore, as trogocytosis and cross-dressing widens the spectrum and increases the flexibility of the self, organism-organism relationship, which also includes host-pathogen interactions, are also largely affected by the trogocytosis-associated phenomena.

Although comparative genomics and proteomics are powerful tools to elucidate molecular mechanisms of the ubiquitous core units of biological processes, central components of phagocytosis ubiquitous in eukaryotes have not been identified with an exception of actin, by phagosome proteomics of 5 eukaryotic organisms [155]. This important observation indicates that the molecular evolution of trogocytosis cannot be elucidated only by multitaxa comparison of orthologous genes known to be involved in trogocytosis. Such presumption prompts us to investigate lineage-specific molecular mechanisms of trogocytosis in each system based on the shared morphological and molecular annotation/definition of trogocytosis. Once trogocytosis is demonstrated and mechanistically characterized in a broader range of unicellular organisms including free-living protists, trogocytosis may gain full attention as a ubiquitous mechanism to acquire molecules and associated information from other organisms in the environment. Such paradigm shift has tremendous impact on our understanding of eukaryote evolution.

## Figures and Tables

**Table 1:** Trogocytosis by unicellular eukaryotes. List of unicellular protozoa in which trogocytosis or a trogocytosis-like process was demonstrated.

Trogocyte	Life Style	Life Cycle	Trogocytosis Target	References
*Amoeba proteus*	free-living	trophozoite	ciliate (Paramecium, Frontonia)	[3,4]
*Chaos carolinensis*	free-living	trophozoite	ciliate (Blapharisma)	[5]
*Dictyostelium caveatum*	free-living	trophozoite	other Dictyosterium (D. discoideum)	[6,7]
*Naegleria fowleri*	free-living/parasititc (extracellular)	trophozoite	mammalian cell	[8,9,10]
*Harmannella-Acanthamoeba*	free-living/parasititc (extracellular)	trophozoite	mammalian cell	[8,9]
*Entamoeba histolyca*	extracellular parasite (intestine)	trophozoite	mammalian cell	[16,17]
*Teichomonas vaginalis*	extracellular parasite (urogenital tract)	trophozoite	mammalian cell	[18,19]
*Plamodium falciparum*	intracellular parasite (erythrocyte)	erythtocytic (trophozoite)	parasitizing erythrocyte	[20,21,22,23]
*Toxoplasma gondii*	intracellular parasite (nucleated cells)	tachyzoites; bardizoites	parasitizing mammalian cell	[24]

**Table 2:** Proteins involved in trogocytosis and phagocytosis in *E. histolytica*. See Figure 1 for details.

Category	Molecules	Trogocytosis	Phagocytosis
Adherence	Surface Molecules	Gal lectin	Gal lectin Transmembranare kinases SREHP/Ariel EHI_098510 EhGPCR-1
Signaling	Phosphatidylinositol kinases	PI3K	PIPKI PI3K
	Protein kinases	C2PK AGCK1 AGCK2	C2PK AGCK2 AK1
	Calcium binding proteins	Unknown	CaBP1/3/5
Formation	Cytoskeletal proteins and regulators	actin	Actin Formin Arp2/3 complex EhRho1 EhRacA
Closure	Cytoskeletal proteins and regulators	Unknown	Myosin IB
Maturation	Phospholipids, vesicular traffic-related proteins	PtdIns3P Vps26 (retromer) Atg8	PtdIns3P Vps26 (retromer) Atg8
